# Deep sequencing reveals as-yet-undiscovered small RNAs in *Escherichia coli*

**DOI:** 10.1186/1471-2164-12-428

**Published:** 2011-08-24

**Authors:** Atsuko Shinhara, Motomu Matsui, Kiriko Hiraoka, Wataru Nomura, Reiko Hirano, Kenji Nakahigashi, Masaru Tomita, Hirotada Mori, Akio Kanai

**Affiliations:** 1 Institute for Advanced Biosciences, Keio University, Tsuruoka 997-0017, Japan; 2 Systems Biology Program, Graduate School of Media and Governance, Keio University, Fujisawa 252-0882, Japan; 3 Graduate School of Biological Sciences, Nara Institute of Science and Technology, 8916-5 Ikoma, 630-0192, Japan; 4 Faculty of Environment and Information Studies, Keio University, Fujisawa 252-0882, Japan

## Abstract

**Background:**

In *Escherichia coli*, approximately 100 regulatory small RNAs (sRNAs) have been identified experimentally and many more have been predicted by various methods. To provide a comprehensive overview of sRNAs, we analysed the low-molecular-weight RNAs (< 200 nt) of *E. coli *with deep sequencing, because the regulatory RNAs in bacteria are usually 50-200 nt in length.

**Results:**

We discovered 229 novel candidate sRNAs (≥ 50 nt) with computational or experimental evidence of transcription initiation. Among them, the expression of seven intergenic sRNAs and three *cis*-antisense sRNAs was detected by northern blot analysis. Interestingly, five novel sRNAs are expressed from prophage regions and we note that these sRNAs have several specific characteristics. Furthermore, we conducted an evolutionary conservation analysis of the candidate sRNAs and summarised the data among closely related bacterial strains.

**Conclusions:**

This comprehensive screen for *E. coli *sRNAs using a deep sequencing approach has shown that many as-yet-undiscovered sRNAs are potentially encoded in the *E. coli *genome. We constructed the *Escherichia coli *Small RNA Browser (ECSBrowser; http://rna.iab.keio.ac.jp/), which integrates the data for previously identified sRNAs and the novel sRNAs found in this study.

## Background

RNA molecules are known to be key genetic regulators in diverse organisms. In bacteria, these regulatory RNAs are generally referred to as small RNAs (sRNAs) because they usually range from 50 to 200 nt in length [1]. Recent studies have suggested that most sRNAs modulate target gene expression at the post-transcriptional level by base pairing to mRNAs [2]. It has been reported that the majority of bacterial sRNAs are synthesised under very specific growth conditions and that these RNAs are regulators of gene expression in response to environmental stresses [2,3] such as low iron [4], oxidative stress [5] and elevated glucose-phosphate levels [6]. Currently, approximately 400 sRNAs have been detected in 70 microbial genomes, including of the *Escherichia*, *Shigella *and *Salmonella *genera [1]. In *Escherichia coli *(*E. coli*), the most exhaustive and diverse searches for sRNAs have been conducted with several methods: high-throughput computational searches [7-13], shotgun cloning [14,15] and tiling array analyses [16,17]. As a result, 80 sRNAs have been experimentally verified and registered in RegulonDB [18], and many more sRNAs are predicted to exist [19]. In recent years, deep sequencing has emerged as a new and powerful experimental method for transcriptome analysis [20]. In eukaryotic organisms, this approach has been commonly used for transcriptome analyses, including microRNA detection [21]. In contrast, in bacteria, this approach has only recently been used for transcriptome analysis, although it has the potential to increase our insight into transcriptional and post-transcriptional events in microorganisms dramatically [20-22]. For instance, several sRNAs have been identified using a deep sequencing-based approach in *Salmonella *[23,24], *Vibrio cholerae *[25], *Helicobacter pylori *[26], *Burkholderia cenocepacia *[27], *Bacillus anthracis *[28] and quite recently, *E. coli *[29].

In this study, we report novel sRNAs identified in *E. coli *with a deep sequencing analysis, focusing on low-molecular-weight RNAs (< 200 nt). Applying this approach, we successfully detected most of the previously known sRNAs, and discovered thousands of novel (fragmentary) transcribed regions. We selected 229 novel transcribed regions as candidate sRNAs with computational or experimental evidence of transcription initiation. Furthermore, we detected the expression of seven intergenic sRNAs and three *cis*-antisense sRNAs by northern blot analysis. Interestingly, five newly identified sRNAs are expressed from prophage regions. We conducted an evolutionary conservation analysis of our candidate sRNAs and summarised the data among closely related bacterial strains. Finally, we generated a platform (*Escherichia coli *Small RNA Browser; ECSBrowser) for sRNA research in bacteria.

## Results

### Deep sequencing-based identification of *cis*-antisense and intergenic sRNAs

**Figure 1:**
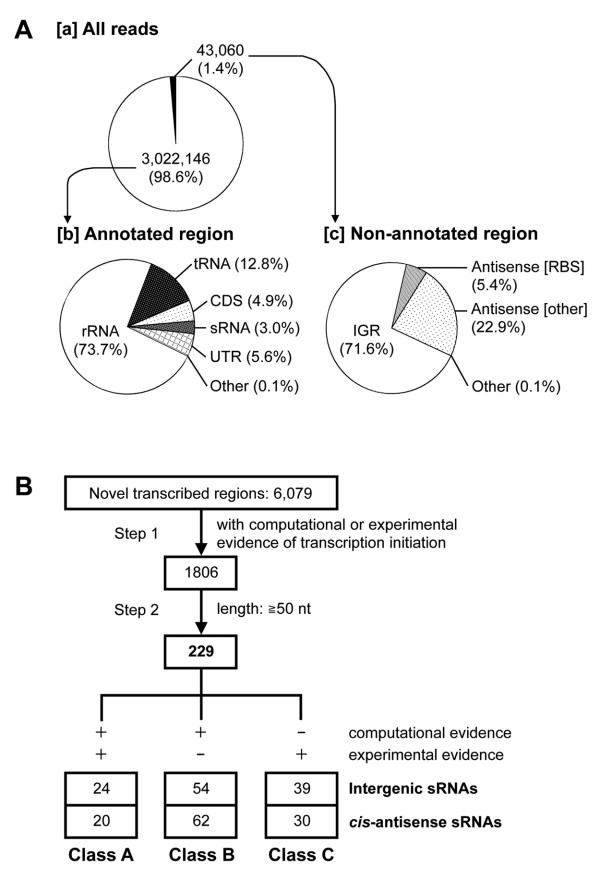
**Summary of the deep sequencing analysis of low-molecular-weight RNAs and the extraction of novel sRNAs in *E. coli***. (A) Pie charts classifying the deep sequencing reads. [a] The percentages of all the deep sequencing reads mapped to the previously annotated or non-annotated regions of the *E. coli *genome. [b] The relative proportions of the deep sequencing reads (*n *= 3,022,146) mapped to the annotated regions. 'Other' includes the reads that mapped to pseudogenes or phantom genes. The reads that mapped to several genes of various types were also classified as 'Other'. [c] The relative percentages of the deep sequencing reads (*n *= 43,060) mapped to the non-annotated regions. IGR, intergenic region; Antisense [RBS], reads located on the *cis*-antisense strand of the ribosomal binding site of a known gene; Antisense [other], reads located on the *cis*-antisense strand of a known gene; Other, reads mapped to both intergenic and *cis*-antisense regions. (B) Schematic representation of the procedure used to extract and classify novel candidate sRNAs. Novel candidate sRNAs were extracted from all novel transcribed regions by the following two steps: selection of transcribed regions with evidence of transcription initiation (Step 1) and length (Step 2). Then, novel candidate sRNAs were classified according to their evidence for transcription initiation and their coding positions in the *E. coli *genome; Class A, with both computationally predicted sigma 70 promoters and experimentally determined TSSs [30-33] and/or RBRs [30]; Class B, with only computationally predicted sigma 70 promoters; Class C, with only experimentally determined TSSs and/or RBRs.

To comprehensively identify and summarise the sRNAs in *E. coli*, we focused on the low-molecular-weight RNAs and performed a deep sequencing analysis. First, *E. coli *cells were grown to late exponential phase in M63 medium (glucose minimal medium). The cultured cells were harvested and immediately treated with RNAprotect Bacteria Reagent to prevent RNA degradation, because (i) the half life of some sRNAs is very short (< 2 min) [15]; and (ii) we wanted to minimise *in vitro *degradation. The low-molecular-weight RNA (< 200 nt) was then isolated from the cultured cells (Additional File 1A), and a cDNA library was constructed from the RNA sample after it had been ligated to both 5'- and 3'-specific RNA adapters to determine the direction of each transcript. The cDNA library was deep sequenced using an Illumina 1G Genome Analyzer system (a total of 12,473,172 reads). After discarding the reads containing unreliable nucleotides (Additional File 1B, steps 1 and 2), we selected 3,065,206 reads that could be mapped to the *E. coli *genome with SOAP v1 (Additional File 1B, step 3). Most of the reads (98.6%) were mapped to annotated regions, whereas the remaining 43,060 reads (1.4%) were mapped to non-annotated regions (Figure 1A [a]). The majority of reads that mapped to annotated regions (Figure 1A [b]) corresponded to rRNAs (73.7%) or tRNAs (12.8%), and the remainder corresponded to coding sequences (CDSs; 4.9%), previously known sRNAs (3.0%), untranslated regions (UTRs; 5.6%) or other regions such as pseudogenes or phantom genes (0.1%). Conversely, the reads that mapped to the non-annotated regions (Figure 1A [c]) corresponded to either intergenic regions (71.6%) or *cis*-antisense strands of known genes (28.3%). We classified the reads that could be mapped to both intergenic regions and *cis*-antisense strands of known genes as "other regions" (0.1%). The reads located on *cis*-antisense strands of the ribosomal binding sites (RBSs) of known genes, in particular, may be involved in post-transcriptional regulation of mRNAs [2]. We noted that 2,326 reads (5.4% of the total) were classified in this category.

To predict novel transcribed regions, the resulting reads were classified into one of five groups (Groups A-E), according to the numbers (single or multiple) and types (annotated or non-annotated) of the mapped regions (Additional File 1B, step 4). We then eliminated the non-annotated mapped regions in Group E, because the reads in Group E matched multiple regions and because it was difficult to determine which one(s) was actually expressed. Finally, all overlapping mapped regions were assembled, thus generating 6,079 novel transcribed regions and 16,895 known transcribed regions (Additional File 1B, step 5). We assumed that some of these regions were still fragmentary. It is noted that 67 of 80 known sRNAs registered in RegulonDB 6.3 were detected with multiple deep sequencing reads, indicating the reliability of our method. The remaining 13 known sRNAs have been reported to be expressed only under specific conditions (Additional File 2).

Then, we attempted to extract novel sRNAs from the 6,079 novel transcribed regions. For this purpose, we focused on novel transcription units. Generally, sRNAs can be generated either as primary transcripts or by processing from longer precursor transcripts [15]. In this study, we ignored the latter case because we could not discriminate the processed sRNAs from RNA degradation products in the current experiment. We used both computational and experimental data to reveal the novel transcriptional units. Firstly, we searched for potential promoter and terminator sequences at the genome level, as described previously [9], and predicted 47,630 sigma 70 promoter sequences and 5,290 rho-independent terminator sequences. The observed-to-expected (O/E) ratio of the predicted sigma 70 promoters for the 6,079 novel transcribed regions was lower than that for the 741 known genes with annotated sigma 70 promoters (Additional File 3A), but it is striking that their undulating patterns were very similar. These results suggest that many of the novel transcribed regions contain predicted sigma 70 promoters, as do the known genes. However, in the case of rho-independent terminators, the O/E ratio of the 6,079 novel transcribed regions was not significant (Additional File 3B), suggesting that most of the novel transcribed regions may not have a defined transcription termination site or may lack the 3' sequence. Based on these results, we decided to use only the transcription initiation information to extract the candidate sRNAs. Next, we collected published data on experimentally determined transcription start sites (TSSs) [30-33] and RNA polymerase-binding regions (RBRs) [30]. Finally, we selected 229 novel transcribed regions as candidate sRNAs. The criteria for this selection process were as follows (a flow chart is shown in Figure 1B): (i) computational or experimental evidence of transcription initiation; and (ii) a length of more than 50 nt. These candidate sRNAs were designated ECS001-229. Of these candidate sRNAs, 117 were located in intergenic regions (intergenic candidate sRNAs) and the remaining 112 were located on the opposite strand of annotated regions (*cis*-antisense candidate sRNAs). We also classified these 229 candidate sRNAs into three classes (A-C) according to the evidence for transcription initiation (Figure 1B). The details of these candidate sRNAs are summarised in Additional File 4.

It has been reported that small functional proteins of less than 50 amino acids [34,35] and several regulatory sRNAs encode small functional peptides (so called "dual-function sRNAs") in bacteria [36,37]. Therefore, we scanned the sequence of each candidate sRNA for an open reading frame (ORF) and corresponding RBS using a computational approach, to determine whether our novel candidate sRNAs could encode small peptides. We determined that 29 candidate sRNAs had both an ORF and a corresponding RBS, suggesting that these sRNAs may encode small peptides (Additional File 5A, Type 1 & Additional File 5B). Seventy-five candidate sRNAs had putative start codons and corresponding RBSs, although they did not have stop codons (Additional File 5A, Type 2). Fifty-five candidate sRNAs had putative ORFs, although they did not have corresponding RBSs (Additional File 5A, Type 3). Collectively, our data suggest that additional small proteins might yet be discovered among these candidate sRNAs. In contrast, the remaining 70 candidate sRNAs did not have ORFs of an appropriate length or RBSs, suggesting that these function as non-coding RNAs (ncRNAs). The details of these candidate sRNAs are summarised in Additional File 4.

### Expression of novel sRNAs analysed by northern blot hybridisation

Deep sequencing analysis revealed a large number of novel transcribed regions, identifying 229 intergenic and *cis*-antisense candidate sRNAs. We used northern blot analysis to confirm the expression and determine the sizes of these sRNAs, with strand-specific probes for the 25 most abundant candidate sRNAs with predicted sigma 70 promoters (ECS001-025; Additional File 4). Sixteen of these were intergenic sRNAs and nine were *cis*-antisense sRNAs. We used both total RNA and low-molecular-weight RNA from *E. coli *cells grown to either exponential or stationary phase. We defined the expected size of each sRNA as the length of the continuous genomic region mapped with at least one read.

#### Intergenic sRNAs

**Figure 2:**
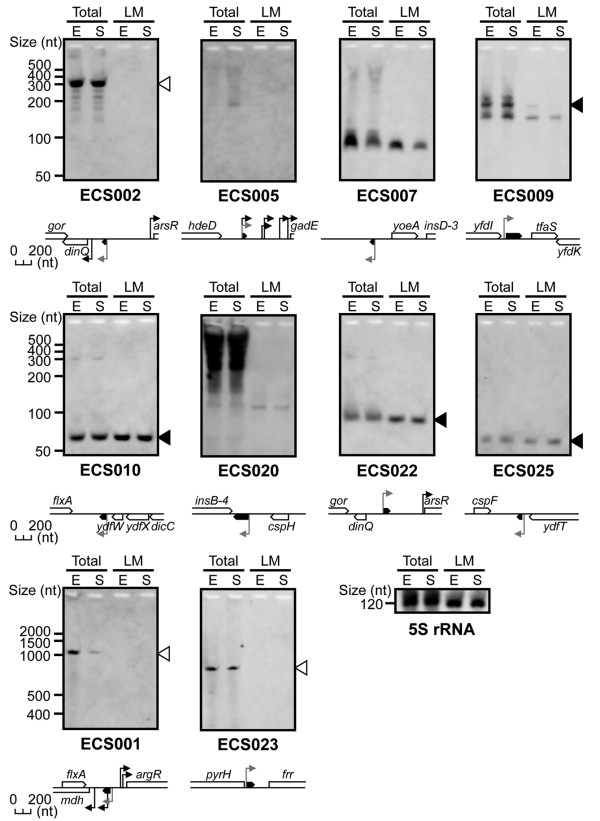
**Identification of novel intergenic sRNAs with sigma 70 promoters using northern blot analysis**. The expression of ten transcripts from intergenic regions is shown (these candidates were selected from classes A and B; Figure 1B). The genomic locations of the sRNA regions (black boxed arrows) and the adjacent genes (white boxed arrows) are shown below each panel (black arrow, known promoter; grey arrow, predicted sigma 70 promoter for each sRNA). RNA samples were isolated from *E. coli *cells grown to exponential (E) or stationary (S) phase in M63 minimal medium. Total, total RNA (20 μg per lane); LM, low-molecular-weight RNA (2 μg per lane). Black triangles indicate that the band size was approximately the same as the predicted length of the sRNA based on the deep sequencing analysis. White triangles indicate bands that may represent new TSSs for downstream genes. 5S rRNA expression is shown as the loading control.

Expression of 10 of the 16 intergenic candidate sRNAs was detected by northern blot analysis (Figure 2) and the results are summarised in Table 1. The lengths of each main band on the ECS009, ECS010, ECS022 and ECS025 blots were almost the same as the expected sizes (indicated with a black triangle). For the remaining six candidate sRNAs (except ECS020), the observed size was longer than the expected size. Among them, the observed sizes of ECS001, ECS002 and ECS023 were almost equivalent to the distance between the 5' end of each candidate sRNA and the 3' end of the nearest downstream gene. Based on the RegulonDB database and the results of this study, the longer transcripts may be expressed from novel TSSs (*i.e*., each transcript detected by deep sequencing analysis was part of a 5'-UTR, not an sRNA). For three candidate sRNAs, ECS005, ECS007 and ECS020, the reason for the difference between the observed band size and the expected band size remains unknown. Although the band for ECS005 was very faint on northern blots, we obtained a similar expression pattern using two different probes (data not shown), and thought that the candidate ECS005 was a member of the novel sRNA category. For ECS020, we detected a smear in the total RNA lanes on northern blots and eliminated this candidate from being a novel sRNA because we did not detect a similar expression pattern using two different probes (data not shown). Intriguingly, the ECS001, ECS005 and ECS007 sRNAs were differentially expressed in the four different growth phases (Additional File 6), suggesting that the expression of these RNAs is regulated during bacterial growth.

**Table 1:** The 16 most abundant intergenic candidate sRNAs with predicted sigma 70 promoters

sRNA name	Size (nt)	Mean no. of Reads*^a^	Northern blot band size*^b ^**(nt)**		Ref.*^c^
				
			Total	LM	
ECS001	89	872.9	~1130	**-**	-
ECS002	51	360.4	~300	**-**	[16]
ECS004	78	306.0	**-**	**-**	[11]
ECS005	62	197.9	~190	**-**	**-**
ECS006	89	83.3	**-**	**-**	**-**
ECS007	50	79.5	~90	~90	**-**
ECS009	186	52.0	**~190**, ~155	~155	[9,13,17]
ECS0010	74	47.5	**~75**	**~75**	[9]
ECS0014	107	31.0	**-**	**-**	**-**
ECS0016	73	24.8	**-**	**-**	**-**
ECS0019	66	19.6	**-**	**-**	**-**
ECS0020	179	18.8	smear	~115	**-**
ECS0022	79	17.1	**~80**	**~80**	[16]
ECS0023	87	16.7	~840	**-**	**-**
ECS0024	74	14.9	**-**	**-**	**-**
ECS0025	60	14.5	**~65**	**~65**	**-**

Sixteen intergenic candidate sRNAs were extracted from classes A and B (Figure 1B) for northern blot analysis. *^a ^The average number of deep sequencing reads mapped to each candidate sRNA: total number of reads divided by the length of the candidate sRNA, to compare the relative expression levels. *^b ^Major signal sizes detected by northern blotting (Figure 2). Total, total RNA; LM, low-molecular-weight RNA. Bold letters indicate that each signal size was approximately the same as the predicted length of the sRNA based on the deep sequencing analysis. Hyphens indicate that no signal was detected in the northern blot analysis. *^c ^References to predicted sRNAs in previous studies located around each candidate sRNA. The details of the candidate sRNAs are given in Additional File 4.

#### *Cis*-antisense sRNAs

**Figure 3:**
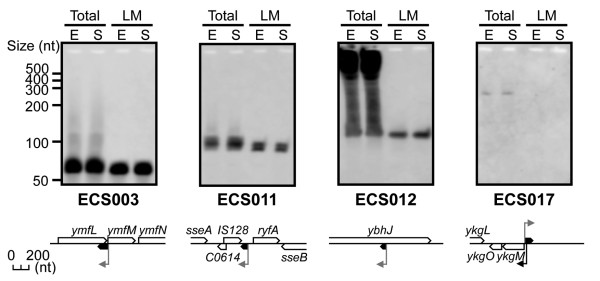
**Identification of novel *cis*-antisense sRNAs with predicted sigma 70 promoters using northern blot analysis**. The expression of four *cis*-antisense sRNAs is shown (these candidates were selected from classes A and B; Figure 1B). The genomic locations of the sRNA regions and the adjacent genes are shown below each panel, as in Figure 2. See Figure 2 for the RNA samples.

The expression of four of the nine *cis*-antisense candidate sRNAs was detected by northern blot analysis (Figure 3) and the results are summarised in Table 2. The observed size of each candidate sRNA differed slightly from the expected size. However, for the ECS003 candidate sRNA, the length of the mapped region with many reads (78 nt) was consistent with the observed size (~70 nt). A faint band was also observed at ~120 nt, which corresponded to the expected size. These results suggest that ECS003 is first transcribed as a precursor of approximately 120 nt and is then cleaved to ~70 nt. The observed sizes of the remaining candidate sRNAs were somewhat longer than expected (Table 2).

**Table 2:** The nine most abundant *cis*-antisense candidate sRNAs with predicted sigma 70 promoters

sRNA name	Size (nt)	Mean no. of Reads*^a^	Putative target genes*^b^		Northern blot band size^*c ^(nt)		Ref.*^d^
				
			Name	Product	Total	LM	
ECS003	116	357.9	*ymfL & ymfM*	e14 prophage; predicted protein	~70	~70	-
ECS008	75	53.4	*yfaS_1*	pseudogene; predicted protein	ND	ND	-
ECS0011	71	46.5	*IS128*	small RNA (unknown function)	~95	~95	[9]
					~100	~100	
ECS0012	66	39.6	*ybhJ*	predicted hydratase	smear	~120	-
ECS0013	67	38.1	*rumA*	23S ribosomal RNA 5-methyluridine methyltransferase	ND	ND	-
ECS0015	54	25.7	*nudC*	NADH pyrophosphatase	ND	ND	-
ECS0017	92	22.3	*ykgM *&*ykgO*	predicted ribosomal protein	~275	ND	[11]
ECS0018	90	21.6	*gltBDF *operon	glutamate synthase & periplasmic protein	ND	ND	[9,13]
ECS0021	57	17.6	*ytfP *&*ytfN*	conserved protein	ND	ND	-

Nine *cis*-antisense candidate sRNAs were extracted from classes A and B (Figure 1B) for northern blot analysis. *^a ^The average number of deep sequencing reads mapped to each candidate sRNA: total number of reads divided by the length of the candidate sRNA. *^b ^The names and products of the sense-strand genes (putative target genes) corresponding to each *cis-*antisense candidate sRNA. *^c ^Major signal sizes detected by northern blotting (Figure 3). Total, total RNA; LM, low-molecular-weight RNA. Hyphens indicate that no signal was detected in the northern blot analysis. *^d ^References to predicted sRNAs in previous studies located around each candidate sRNA. The details of the candidate sRNAs are given in Additional File 4.

It is well known that *cis*-antisense sRNAs and their target mRNAs exhibit complete complementarity, and that their base-pairing causes either translational regulation of the target RNA by changing the ratio of its ribosomal-binding activity or degradation of the target RNA by certain ribonuclease(s) [2]. Furthermore, it has been reported that a subset of sRNAs regulates transcription [38]. For example, the ECS003 sRNA is encoded on the *cis*-antisense strand of the *ymfM *RBS, and might regulate *ymfM *translation by masking the RBS. The ECS017 sRNA is encoded on the *cis*-antisense strand of the sigma 70 promoter (ykgMp) of the *ykgM-ykgO *operon and the ECS012 sRNA is encoded on the *cis*-antisense strand of the *ybhJ *mRNA (protein-coding region), suggesting that these *cis*-antisense sRNAs might regulate their corresponding sense transcript. In contrast, the ECS011 sRNA is encoded on the *cis*-antisense strand of the IS128 sRNA. It has previously been reported that the IS128 sRNA overlaps the IS129 sRNA in the same chromosomal region as a *cis*-antisense sRNA pair [9]. Both the IS128 and IS129 sRNA transcripts (90-95 nt) were detected by northern blot analysis with a double-stranded probe that could not discriminate between the strands. We found that only IS128 is registered in the RegulonDB version 6.3 database. However, in our study, no IS128 sRNA was detected by northern blotting with strand-specific probes (data not shown), although IS129 was detected as part of ECS011. Therefore, we suggest that the IS128 sRNA is an artefact or is only expressed under specific conditions. We also suggest a re-annotation of IS129 as ECS011, a member of the intergenic sRNAs.

### Novel sRNAs expressed from prophage regions and their possible roles

The *E. coli *K12 genome is reported to include 10 prophages [39]. We noted that 27 of 229 candidate sRNAs were expressed from prophage regions (Additional File 4). To date, the *isrC *[9], *C0293 *[16] and *dicF *[40] sRNAs are known to be expressed from prophage regions. The functions of the *isrC *and *C0293 *sRNAs are totally unknown, although the *dicF *sRNA is known to bind the RBS of the *ftsZ *mRNA, thus inhibiting ribosome entry. Because *ftsZ *encodes a protein (Z ring) involved in cell division, *dicF *sRNA binding causes a defect in *E. coli *cell division [41]. To investigate our sRNAs expressed from prophage regions, we focused on five sRNAs detected by northern blot analysis (Figures 2 and 3).

**Figure 4:**
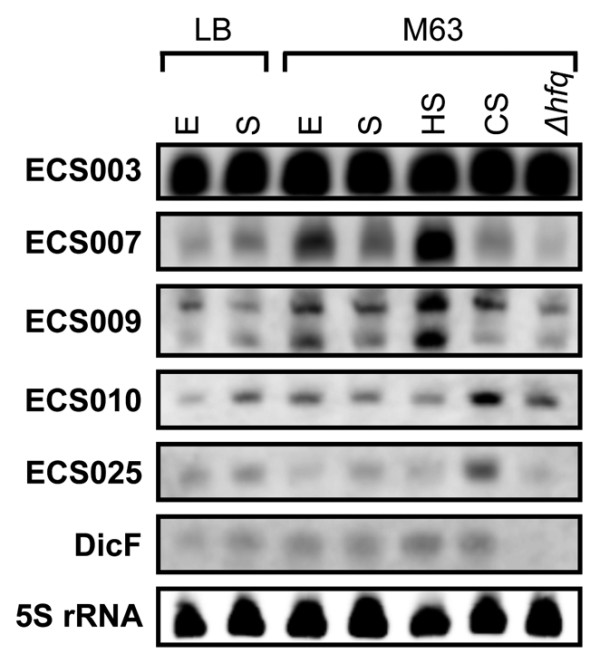
**Northern blot analysis of five novel sRNAs in prophages**. Total RNA was isolated from *E. coli *cells grown to exponential (E) or stationary (S) phase in LB medium or M63 minimal medium and from cells subjected to heat shock (HS) or cold shock (CS). Total RNA was also isolated from the *hfq *knockout strain grown to exponential phase in M63 minimal medium (*Δhfq*). 5S rRNA expression is shown as the loading control.

Because the expression of many sRNAs is reported to be regulated under specific conditions [2,3], such as under various stresses, in different kinds of media or in the presence of certain chemicals, we analysed the expression of each sRNA expressed from a prophage region using total RNAs prepared from cells grown under these conditions (Figure 4). We used total RNAs from *E. coli *cells grown to exponential or stationary phase in LB (or M63) medium or *E. coli *cells subjected to heat or cold shock. We also used an *hfq *knockout strain (grown to exponential phase in M63 medium) because the RNA chaperone Hfq is thought to be required for the stability of many sRNAs and/or their activity in *E. coli *[42]. We measured the signal intensity of each band using the Quantity One software package (Bio-Rad Laboratories), and calculated the relative amount of each transcript by comparison with the signal intensity of that transcript in samples derived from cells grown to exponential phase in M63 medium, which were used as the control. The gene expression of the ECS003 sRNA did not change under any of these conditions, although its transcript level seemed to be relatively stronger. In contrast, the levels of the ECS007 and ECS010 transcripts were reduced (approximately 0.2-0.3-fold) in LB medium (lane E in Figure 4). The expression of the ECS007 and ECS009 sRNAs was increased (approximately 1.5-2-fold) by heat shock, whereas that of the ECS010 and ECS025 sRNAs was increased (approximately 2-3-fold) by cold shock. The levels of the ECS007 and ECS009 transcripts were reduced (approximately 0.2-0.5-fold) in the *hfq *knockout strain. The expression of *dicF *sRNA, which is known to bind Hfq, was also reduced and was negligible in the *hfq *knockout strain. Moreover, the ECS009 transcript overlapped with previously predicted candidate sRNAs that have been detected based on co-immunoprecipitation with Hfq [17] (Table 1). These results suggest that Hfq is required to produce or maintain the ECS007 and ECS009 sRNAs.

### Nucleotide conservation of novel candidate sRNAs in bacteria

**Figure 5:**
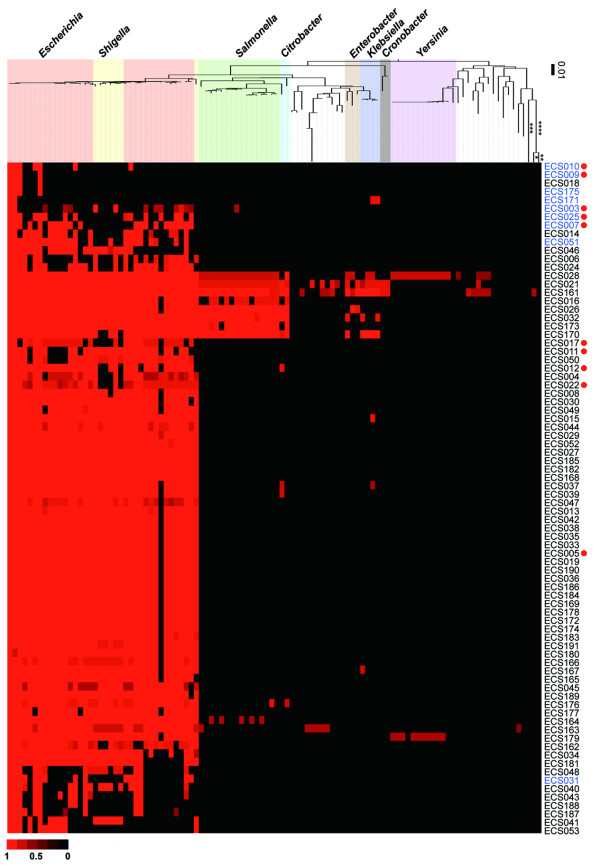
**Nucleotide conservation of novel sRNAs in bacteria**. The nucleotide sequence conservation of the 80 most abundant candidate sRNAs among 106 closely related bacterial strains is shown. Red indicates high nucleotide conservation and black indicates no conservation. The name of each sRNA is shown on the right. sRNAs expressed from prophage regions (blue letters) and sRNAs identified by northern blot analysis (red circles) are indicated. The phylogenetic tree for 16S rRNA sequences was constructed based on the neighbour-joining method using ClustalW version 1.83 [64]. The names of the organisms and their corresponding NCBI IDs for the complete genome sequence are described in Additional File 10. Evolutionary distance is shown by the branch lengths in the phylogenetic tree. For three strains, the lines have been shortened (the real evolutionary distances are *, 0.49; **, 0.48; ***, 0.07; and ****, 0.42).

The Blast-Like Alignment Tool (BLAT) [43] was used to compare the nucleotide sequences of the 80 previously known sRNAs and 229 novel candidate sRNAs in *E. coli *with those of 1,378 complete bacterial genomes. Under our definition of "conservation" (see the Methods section), we found that only 106 genomes derived from 22 genera possessed similar sequences, and that most of the conservation was restricted to two genera: *Escherichia *and *Shigella*. An example of the nucleotide conservation of the 80 most abundant candidate sRNAs is shown in Figure 5. The same features of conservation were reported in a previous study using 55 sRNAs [19]. In contrast, some novel sRNAs, such as ECS016 and ECS173, exhibited conservation with sequence from the *Salmonella *genus. Moreover, the ECS021, ECS028 and ECS161 sRNAs were conserved beyond the *Yersinia *genus, similar to housekeeping sRNAs such as *ssrS *sRNA (6S RNA) and *ffs *sRNA (4.5S RNA). sRNAs expressed from prophage regions, such as ESC009, ECS010 and ECS171, were not well conserved even within the *Escherichia *genus. Because the host range of some bacteriophages is quite limited and, in many cases, each bacteriophage only attacks a single strain of bacterium [44], the prophage region itself may not demonstrate high levels of nucleotide conservation in the genome.

### Construction of the ECSBrowser

**Figure 6:**
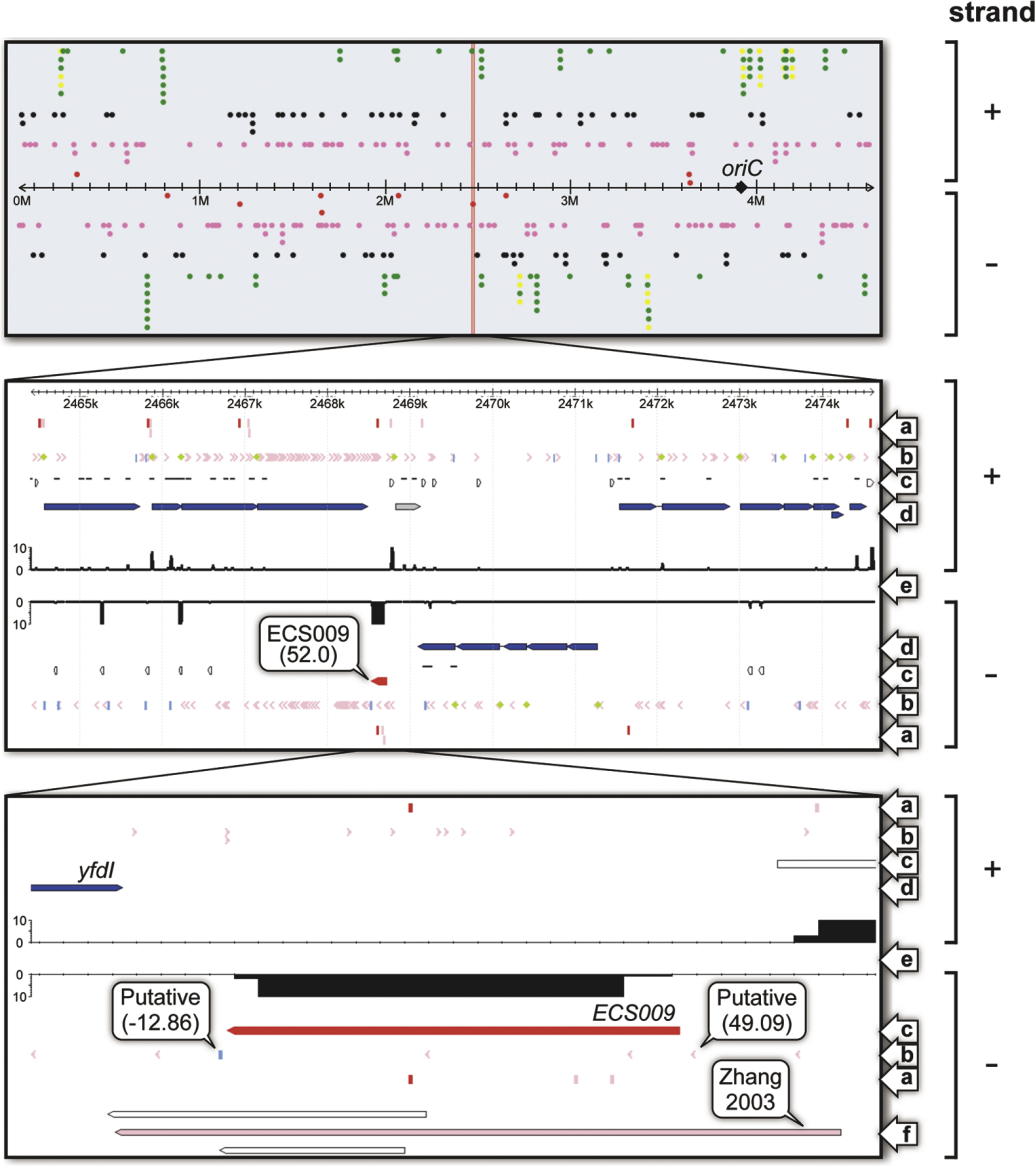
***Escherichia coli *Small RNA Browser**. Three screen shots are shown as examples of the *Escherichia coli *Small RNA Browser (ECSBrowser; http://rna.iab.keio.ac.jp/). The upper panel shows an overview of all the non-coding RNA genes encoded in the *E. coli *genome: novel sRNAs detected by northern blot analysis (red circles); novel candidate sRNAs (pink circles); previously known sRNAs (black circles); tRNAs (green circles); and rRNAs (yellow circles). The position of the replication origin, *OriC*, is shown as a black rhombus. The middle panel displays the entire CPS-53 prophage. The lower panel focuses on the ECS009 sRNA. Information is given in a balloon when the reader clicks on any of the symbols. For example, if one of the candidate sRNAs is clicked, the name of the candidate sRNA and the average number of deep sequencing reads mapped to that candidate sRNA would appear. The arrows on the right-hand side represent: (a) experimentally determined TSSs (pink vertical lines) and RBRs (red vertical lines) obtained from the published literature [30-33]; (b) consensus sequences of the predicted promoters (pink arrowheads), predicted terminators (blue vertical lines) and predicted RBSs (green rhombi); (c) novel sRNAs detected by northern blot analysis (red boxed arrows), novel transcribed regions (white boxed arrows) and known transcribed regions (black lines); (d) CDSs (blue boxed arrows) and pseudogenes (grey boxed arrows); (e) deep sequencing data; and (f) sRNAs predicted by computational methods (white boxed arrows) and sRNAs predicted by experimental procedures (pink boxed arrows) in previous studies.

We constructed the ECSBrowser to provide an up-to-date overview of *E. coli *sRNAs, including those identified in this study (Figure 6). This database enables visualisation of all the data from the current analysis. In Figure 6, the upper panel shows all the ncRNA genes encoded in the *E. coli *genome, including sRNAs, tRNAs and rRNAs. The middle panel magnifies the entire CPS-53 prophage. The lower panel focuses on the ECS009 sRNA. In this figure, we used the example of ECS009, expressed from the CPS-53 prophage region. Similarly, using the ECSBrowser, we can obtain information for each sRNA, from the single-nucleotide level to the whole-genome level. We also included in this browser the evolutionary conservation data for the 80 previously known sRNAs and 229 novel sRNAs. The ECSBrowser is available through the website http://rna.iab.keio.ac.jp.

## Discussion

In this study, our first priority was to detect novel transcribed regions, so we filtered unreliable reads quite strictly and only selected 3,065,206 high-quality reads from the original 12,473,172 reads (Additional File 1B, steps 1-3). We then discovered 6,079 novel (fragmentary) transcribed regions and selected 229 candidate sRNAs (Figure 1B). It is likely that these candidate sRNAs are actually expressed under the conditions used in this study because the deep sequencing-based approach directly determines the cDNA sequence. Traditionally, the expression of previously known sRNAs was ultimately confirmed by northern blot analysis [45]. Therefore, we also conducted northern blot analysis, detected the expression of 14 of the 25 examined candidate sRNAs (Tables 1 and 2, Figures 2 and 3) and identified 10 of them as novel sRNAs. We cannot explain why the remaining 11 candidate sRNAs could not be detected by northern analysis. It might be necessary to optimise the hybridisation conditions for each sRNA. In addition, we could not intrinsically distinguish the novel sRNAs from stable degradation products in our current study. Therefore, we established criteria for extracting sRNAs (Figure 1B). In this way, we selected 229 candidate sRNAs with computational or experimental evidence of transcription initiation and a length of more than 50 nt (Figure 1B). Because several sRNAs generated by processing from larger precursor RNAs have been reported [14,15] and fairly small sRNAs (≤ 50 nt) are known in a few cases [14], the remaining novel transcribed regions might contain additional sRNAs. Collectively, at least 10 previously unreported sRNAs were identified, and the expression of many other candidate sRNAs was implied by this study, although the functions of the vast majority of these candidate sRNAs are not yet clear.

In the last decade, several groups have searched extensively for sRNAs in *E. coli *using computational and experimental approaches [15-23,29]. Therefore, we examined the overlaps between the candidate sRNAs in this study and those identified in previous studies. Forty-seven of the 229 novel candidate sRNAs overlapped with previously predicted sRNAs, but the remaining 182 could not be predicted by traditional approaches. The details are summarised in Additional File 4 and the ECSBrowser. Moreover, we suggested that 29 of the 229 novel candidate sRNAs might encode small proteins because they have both an ORF (≥ 4 amino acids [aa]) and corresponding RBS. Interestingly, 10 out of 29 potential small proteins in this study overlapped with the dataset of small proteins that have been computationally predicted based on sequence conservation and RBS models [46]. We show six examples of the candidate sRNAs that encode putative peptides in Additional File 4.

The primary limitation of most traditional methods of identifying sRNAs is a bias toward intergenic regions [45]. Therefore, it is likely that the previous list of *cis*-antisense sRNAs is far from complete. Currently, 24 of the 80 previously known sRNAs registered in the RegulonDB database are located on the opposite strand from known genes in *E. coli*. Among them, 12 are reported to act as *cis*-antisense sRNAs [14,15,47-51]. These sRNAs interact with the 5'-UTR [14,48,49,51], the 3'-UTR [50] or the whole area [47] of each target mRNA by a base-pairing mechanism. Two sRNAs are known to interact with each other by a *cis*-antisense mechanism [15]. In this study, we found 112 novel *cis*-antisense candidate sRNAs (Figure 1B). We noted that 74 of these (such as ECS012; Figure 3) are encoded on the opposite strand from protein-coding regions, but are not in the 5'- or 3'-UTRs of the mRNAs (Additional File 4). The target sites of a few *trans*-antisense sRNAs are reported to occur in protein-coding regions and these sRNAs cause endonucleolytic mRNA destabilisation rather than the typical inhibition of translational initiation [52], although most *trans*-antisense sRNAs interact with the 5'-UTR of the target mRNA by a base-pairing mechanism, blocking translation initiation [45]. These results suggest that novel *cis*-antisense sRNA species, as well as *trans*-antisense sRNAs, may regulate a coding gene on the opposite strand.

Three previously identified sRNA genes (*isrC*, *C0293 *and *dicF*) are expressed from prophage regions in *E. coli*. Our results show that at least five additional sRNAs are expressed from prophage regions, and that the transcript levels of some of these sRNAs are high (Figure 4). Recently, it was reported that prophage regions contain several sRNAs and that the expression levels of some sRNAs are also high in *Bacillus subtilis *[46]. This may represent a common feature of prokaryotic sRNAs. Four of five prophage-derived sRNAs were modestly activated by temperature stress (Figure 4), although the expression levels of these sRNAs were unchanged under conditions of SOS induction, such as mitomycin C treatment and UV irradiation (data not shown). The SOS response is known to trigger significantly higher induction of prophages [53].

Previously, it was reported that the majority of intergenic sRNAs in *E. coli *regulate the expression of genes in response to environmental stresses, and that these integral elements of the stress response are usually very tightly regulated [2]. In contrast, the novel sRNAs in the current study are constantly expressed under our conditions, although the transcript levels of several novel sRNAs were also moderately activated in response to temperature stress, nutrient limitation and growth phase (Figures 2, 3, 4). To obtain more functional information on the novel sRNAs, we generated single-deletion mutants for 13 novel intergenic sRNA regions and conducted growth analysis of these mutants on rich and minimal medium. However, the growth of these deletion mutants was not significantly different from that of wild-type *E. coli*. Typical data are shown in Additional File 7. We concluded that many of these sRNAs are not necessary for the growth of *E. coli*, although they are clearly expressed. We are now generating mutants from all the sRNAs for functional analysis (to be published separately). Recently, a deep sequencing analysis of novel regulatory RNAs in *E. coli *has been reported [29]. The authors identified 10 new sRNAs and 9 new regulatory leader sequences in the intergenic regions of *E. coli*. We found that four of our sRNAs (ECS026, ECS181, ECS183 and ECS224) overlapped with their new sRNAs, and another four of our sRNAs (ECS028, ECS031, ECS080 and ECS210) overlapped with their new regulatory leader sequences (summarised in Additional File 4). These results also support the concept that there are many as-yet-undiscovered sRNAs or transcribed regions in *E. coli*.

Finally, we emphasise that the ECSBrowser is the only browser that specifically focuses on the transcribed regions of the *E. coli *genome (Figure 6). This browser includes information about transcript elements, such as promoters, terminators, RBRs and TSSs, which have been predicted and/or identified at the genome-wide level by various methods. Furthermore, this browser includes information about sRNAs that were predicted in eleven previous papers [7,9-17,29]. Using this browser, it is also possible to integrate a number of deep sequencing data that have been obtained under various culture conditions; we intend to use this to compare the dynamic changes in the transcriptome under each specific culture condition. We anticipate that the ECSBrowser will contribute to future transcriptome analysis, including the detection of novel sRNAs.

## Conclusions

In previous research, many bacterial sRNAs have been characterised under certain stress conditions such as heat shock, cold shock or oxidative stress. In this paper, we comprehensively analysed sRNAs expressed in normal *E. coli *growth conditions and discovered 229 novel candidate sRNAs (≥ 50 nt) with computational or experimental evidence of transcription initiation. We also found several novel sRNAs that are highly expressed in prophage regions in the *E. coli *genome. We conducted an evolutionary conservation analysis of the candidate sRNAs and summarised the data among closely related bacterial strains. Furthermore, we made 13 sRNA deletion mutants and characterised their growth, although the growth of these deletion mutants was not significantly different from that of wild-type *E. coli*. Finally, we generated a platform (*Escherichia coli *Small RNA Browser; ECSBrowser) for sRNA research in bacteria. Previously, other studies have reported hundreds of candidate sRNAs encoded in the *E. coli *genome. However, there is no consensus among them and the wealth of information is confusing. In ECSBrowser, users can access the results of the 11 primary *E. coli *sRNA prediction papers plus our new data in a uniform database format. We believe this will be invaluable to researchers in this field.

## Methods

### Data sources

The complete genome sequence of *E. coli *K12 strain MG1655 (NC_000913.2) was downloaded from the National Center for Biotechnology Information (NCBI) Reference Sequence database http://www.ncbi.nlm.nih.gov/RefSeq/. Information on the annotated regions (CDSs, tRNAs, rRNAs, sRNAs, pseudogenes, phantom genes, operons, promoters, terminators and RBSs) was obtained from RegulonDB version 6.3 http://regulondb.ccg.unam.mx/. In this study, we also predicted additional RBSs with RBSfinder using a window size of 50 bp [54], and defined them as an annotated region. Data on experimentally determined TSSs and RBRs were obtained from the literature [30-33].

### Bacterial strains and growth conditions

*E. coli *K12 strain BW25113 (wild type) [55] and the *hfq *knockout strain from the Keio collection [56] were used in this study. *E. coli *was cultured overnight at 37°C under microaerobic conditions in LB medium or modified M63 medium (glucose minimal medium: (K_2_HPO_4 _53.6 g, KH_2_PO_4 _26.2 g, (NH_4_)_2_SO_4 _10.0 g, FeSO_4_.7H_2_O 2.5 mg, D-glucose 4 g, thiamine·HCl 5.0 g and MgSO_4_.7H_2_O 50.0 g per litre). The overnight culture was diluted 20-fold in fresh medium and grown at 37°C to the indicated optical densities at 600 nm (OD_600_). For the heat shock treatment, the cells were grown at 30°C to an OD_600 _of 0.6 and then at 42°C for 15 min. For the cold shock treatment, the cells were grown at 37°C to an OD_600 _of 0.6 and then at 15°C for 30 min. These cultured cells were harvested, immediately treated with RNAprotect Bacteria Reagent (Qiagen, Valencia, CA), according to the manufacturer's protocol, and stored at -80°C until RNA extraction.

### RNA preparation

Two types of RNA samples (total RNA and low-molecular-weight RNA) were prepared from the cultured cells. The total RNA was extracted with an RNeasy Midi Kit (Qiagen), with a slight modification. Instead of using an RNeasy Midi column, we used phenol-chloroform extraction to collect a complete set of RNAs, including low-molecular-weight RNAs. The low-molecular-weight RNA was extracted using the enrichment procedure for small RNAs included in the mirVana™ miRNA Isolation Kit (Ambion, Austin, TX), according to the manufacturer's protocol.

### Deep sequencing of low-molecular-weight RNAs

We used the low-molecular-weight RNAs extracted from *E. coli *K12 strain BW25113 cells grown to an OD_600 _of 0.76 (late exponential phase) for deep sequencing analysis (Additional File 1A). A cDNA library was prepared and the deep sequencing analysis was performed at the Post Genome Institute (Hongo, Tokyo, Japan). Briefly, the RNA samples were treated with tobacco acid pyrophosphatase to convert the 5' terminus from a triphosphate to a monophosphate. The resulting RNAs were then ligated with 5'- and 3'-specific RNA adapters, reverse transcribed and amplified as described in the manufacturer's protocol. The nucleotide sequences were determined with the Illumina/Solexa 1G Genome Analyzer system (Illumina, San Diego, CA), which generates 35-base reads. The deep sequencing data have been submitted to the DDBJ Sequence Read Archive http://trace.ddbj.nig.ac.jp/dra/index_e.shtml under accession number DRA000221.

### Analysis of the deep sequencing data and prediction of the transcribed regions

We used a series of filters to remove unreliable deep sequencing reads. The flow chart for these filtering approaches is summarised in Additional File 1B. In step 1, reads containing the character 'N' (indeterminable A, T, G or C) were discarded from the total reads. In step 2, reads with low-quality base calls were also discarded based on the GERALD module (Illumina). In step 3, we collected the reads that could be mapped to the *E. coli *genome: (i) the first 32 bases of each read were mapped to the *E. coli *genome with SOAP v1 [57], allowing up to two mismatches; and (ii) the remaining 33rd-35th bases were mapped to the *E. coli *genome by forcing perfect alignments, beginning at the 33rd base and retaining the longest region that could be aligned to the genome. In this study, we defined the "mapped region" as the genomic region that could be mapped by some read(s). To predict novel transcribed regions, we classified the filtered reads into one of five groups (Groups A-E) according to the numbers (single or multiple) and types (annotated or non-annotated) of the mapped regions (Step 4). We eliminated the non-annotated mapped regions in Group E because we could not determine whether these mapped regions were actually expressed. Finally, we defined the "transcribed region" as the minimum continuous mapped region, and all overlapping mapped regions were assembled into novel and known transcribed regions (Step 5). After assembling the mapped regions in step 5, we noticed that some non-annotated mapped regions in Groups B and D were contiguous with the known transcribed regions. Thus, we manually transferred these regions to the "known transcribed regions" category.

### Prediction of novel candidate sRNAs

To characterise the transcribed regions and extract novel candidate sRNAs, we first predicted possible promoter and terminator sequences at the genome level using bioinformatics approaches: (i) the consensus sequences of sigma 70 promoters (-35 and -10 box) were predicted with *pftools*2.3 [58] using a profile of *E. coli *sigma 70 promoters [59]; and (ii) the consensus sequences of rho-independent terminators were predicted with RNAmotif [60] based on information from previous studies [13,61].

We calculated the observed number of predicted sigma 70 promoters and rho-independent terminators for each transcribed region. We also calculated the expected number of predicted sigma 70 promoters and rho-independent terminators for random genomic position sets that were generated 100 times using the Blum-Blum-Shub algorithm obtained from CPAN http://search.cpan.org/. The O/E ratio was obtained by dividing the observed numbers by the average expected numbers. As a positive control, the same analysis was also performed for each known gene with an annotated sigma 70 promoter or rho-independent terminator, obtained from RegulonDB version 6.3.

Candidate sRNAs were extracted from all novel transcribed regions based on the following two criteria (a flow chart is shown in Figure 1B). First, the novel transcribed regions with computational or experimental evidence of transcription initiation were extracted (Step 1). We used predicted sigma 70 promoters as the computational evidence. The definition of a possible promoter was that the -10 box consensus sequence of the sigma 70 promoter occurred within 10 bases upstream of a novel transcribed region. We also employed published data on TSSs [30-33] and RBRs [30] as experimental evidence. We used the data only when the TSS was located within 10 bases upstream or downstream of a novel transcribed region and the RBR was found within 50 bases upstream or 10 bases downstream of a novel transcribed region. Second, the novel transcribed regions that were larger than 50 nt were extracted as novel candidate sRNAs (Step 2). Finally, novel candidate sRNAs were classified according to their evidence for transcription initiation (computational or experimental) and their coding positions in the *E. coli *genome (intergenic or *cis*-antisense). Furthermore, we scanned the sequence of each candidate sRNA for a start codon (AUG, UUG or GUG), stop codon (UAA, UGA or UAG), appropriate reading frame (≥ 4 aa) and RBS.

### Northern blot analysis

Both total RNA and low-molecular-weight RNA were prepared from *E. coli *cells grown to an OD_600 _of either 0.6 (the middle of the exponential phase) or 1.2 (the early stationary phase) in our growth conditions. These RNAs (total RNA, 20 μg per lane; low-molecular-weight RNA, 2 μg per lane) were separated on denaturing 6% polyacrylamide gels containing 8 M urea and transferred onto Hybond-N^+ ^membrane (GE Healthcare, Piscataway, NJ) by electroblotting. The 3' ends of specific oligonucleotide probes (summarised in Additional File 9) were labelled using a Biotin 3' End DNA Labeling Kit (Pierce Biotechnology, Rockford, IL). The membranes were hybridised with these probes in ULTRAhyb-Oligo hybridisation buffer (Ambion) at room temperature. The hybridisation was conducted at 42°C for ECS020 sRNA or 65°C for 5S rRNA. The washing temperature was the same as the respective hybridisation temperature. The non-isotopic blots were visualised using a BrightStar BioDetect Kit (Ambion) using the ECF™ Substrate (GE Healthcare). The images were captured using a Molecular Imager FX Pro (Bio-Rad Laboratories, Hercules, CA).

### Nucleotide conservation analysis of both known and novel candidate sRNAs in bacteria

For the conservation analysis, BLAT v.34 [43] was used to compare the nucleotide sequences of the candidate sRNAs in *E. coli *with those of 1,378 complete bacterial genomes downloaded from the NCBI ftp server (http://ftp.ncbi.nlm.nih.gov; 2011/02/11). An sRNA-encoding gene was considered "conserved" in another organism if the sequence met the following three criteria: (i) an E-value lower than 0.01; (ii) a coverage higher than 70%; and (iii) an identity higher than 70%. Then, the nucleotide sequence conservation score was calculated using the following formula: ((nucleotide match-length)*(nucleotide identity/100))/(nucleotide length of the candidate sRNA). These scores are summarised in Additional File 10. Next, we performed hierarchical clustering from the conservation score of candidate sRNAs using Cluster 3.0 software [62] with centroid linkage, and visualised the results using Java TreeView 1.1.5 [63]. For phylogenetic tree construction, we first extracted each 16S rRNA sequence from their corresponding genomic sequences by BLAT using the *E. coli rrsA *gene (16S rRNA) as the query. Then, a multiple alignment of the 16S rRNA sequences was constructed using Clustal W version 1.83 [64] and the phylogenetic tree was depicted using Interactive Tree Of Life [65].

### Construction of the *Escherichia coli *Small RNA Browser (ECSBrowser)

The database for *E. coli *sRNA was constructed using the Generic Genome Browser version 2.0 of the Generic Model Organism Database project http://gmod.org/wiki/Main_Page, and named the *Escherichia coli *Small RNA Browser (ECSBrowser). This browser provides all the information from the current analysis.

### References for Additional Files

Twenty-one references for the Additional Files are listed in Additional File 11.

## List of abbreviations

aa: amino acid; BLAT: Blast-Like Alignment Tool; CDS: coding sequence; *E. coli*: *Escherichia coli*; NCBI: National Center for Biotechnology Information; ncRNA: non-coding RNA; OD: optical density; O/E: observed-to-expected; ORF: open reading frame; RBR: RNA polymerase-binding region; RBS: ribosomal binding site; sRNA: small RNA; TSS: transcriptional start site; UTR: untranslated region.

## Authors' contributions

AS and MM conducted the bioinformatics analyses. AS, MM, KH, WN, RH and KN conducted the experiments. AS and MM developed the browser. AK supervised the project. HM supported the supervision of the project. AS and AK wrote the manuscript. MM, WN, KN, MT and HM supported the writing of the manuscript. All authors have read and approved the final manuscript.

## Supplementary Material

Additional File 1 **Basic flow chart for the deep sequencing analysis of low-molecular-weight RNAs in *E. coli***. (A) Electrophoresis on a denaturing 6% polyacrylamide gel containing 8 M urea of the low-molecular-weight RNA fractions that were used for the deep sequencing analysis. (B) Prediction procedure for novel transcribed regions based on the deep sequencing data. Click here for file

Additional File 2 **Summary of the 80 previously known sRNAs**. Information regarding the previously known sRNAs was obtained from RegulonDB version 6.3 http://regulondb.ccg.unam.mx/. Click here for file

Additional File 3 **Observed-to-expected (O/E) ratios for the sigma 70 promoter and rho-independent terminator for the novel transcribed regions**. (A) The O/E ratios for the predicted sigma 70 promoter were calculated for the novel transcribed regions and for known genes with annotated sigma 70 promoters obtained from RegulonDB version 6.3, as the positive control. (B) The O/E ratios for the predicted rho-independent terminator were calculated for the novel transcribed regions and known genes with annotated rho-independent terminators obtained from RegulonDB version 6.3, as the positive control. Click here for file

Additional File 4 **The 229 novel candidate sRNAs**. Some characteristics of the 229 novel candidate sRNAs are summarized. Click here for file

Additional File 5 **Candidate sRNAs encoding putative small proteins**. (A) Of the 229 candidate sRNAs, 159 were characterised by their small protein-encoding capacity. (B) Screen ECSBrowser shots for six sRNA regions encoding a putative small protein. Click here for file

Additional File 6 **Northern blot analysis confirming the growth-dependent expression of the ECS001, ECS005 and ECS007 sRNAs**. Total RNA (20 μg per lane) was isolated from *E. coli *cells grown to an OD600 of 0.3, 0.6, 0.9 or 1.2 in M63 minimal medium. 5S rRNA expression is shown as the loading control. Click here for file

Additional File 7 **Growth of deletion mutants for six novel sRNAs**. Wild type (WT, *E. coli *K12 strain BW25113) and single-deletion mutants corresponding to each intergenic sRNA region were grown overnight on either LB (rich medium) or M63 (glucose minimal medium) plates at 37, 42 or 20°C. These single-deletion mutants were systematically generated as described previously and the oligonucleotide primers used for the construction of these mutants are summarised in Additional File 8. Click here for file

Additional File 8 **Oligonucleotides used for the construction of single-deletion mutants**. Two successive PCRs were used to construct a DNA fragment for homologous recombination. First PCR primers: lowercase letters indicate the regions homologous to the pKD13 plasmid for the kanamycin resistance gene amplification and uppercase letters indicate the priming site for the second PCR. Second PCR primers: lowercase letters indicate the regions homologous to the target sRNAs and uppercase letters indicate the sequences that were attached in the first PCR. Click here for file

Additional File 9 **Oligonucleotides used for the northern blot analysis**. The location and sequence of each deoxyribonucleotide is shown. Click here for file

Additional File 10 **List of nucleotide sequence conservation scores of the 80 previously known sRNAs and 229 novel candidate sRNAs**. The sequence conservation score was calculated by the following formula: ((nucleotide match-length)*(nucleotide identity/100))/(nucleotide length of the candidate sRNA). The name and NCBI ID of the sequence of each organism's complete genome are indicated. Click here for file

Additional File 11 **References for Additional Files**. This is a reference list for Additional Files. Click here for file
