# Supplementary material for: Deep sequencing reveals as-yet-undiscovered small RNAs in Escherichia coli
Source: BMC Genomics. 2011 Aug 24;12:428. doi: 10.1186/1471-2164-12-428 (PMC3175480; doi:10.1186/1471-2164-12-428)
Supplement: Additional File 1 — Basic flow chart for the deep sequencing analysis of low-molecular-weight RNAs in E. coli. (A) Electrophoresis on a denaturing 6% polyacrylamide gel containing 8 M urea of the low-molecular-weight RNA fractions that were used for the deep sequencing analysis. (B) Prediction procedure for novel transcribed regions based on the deep sequencing data. [file 1471-2164-12-428-S1.PDF]

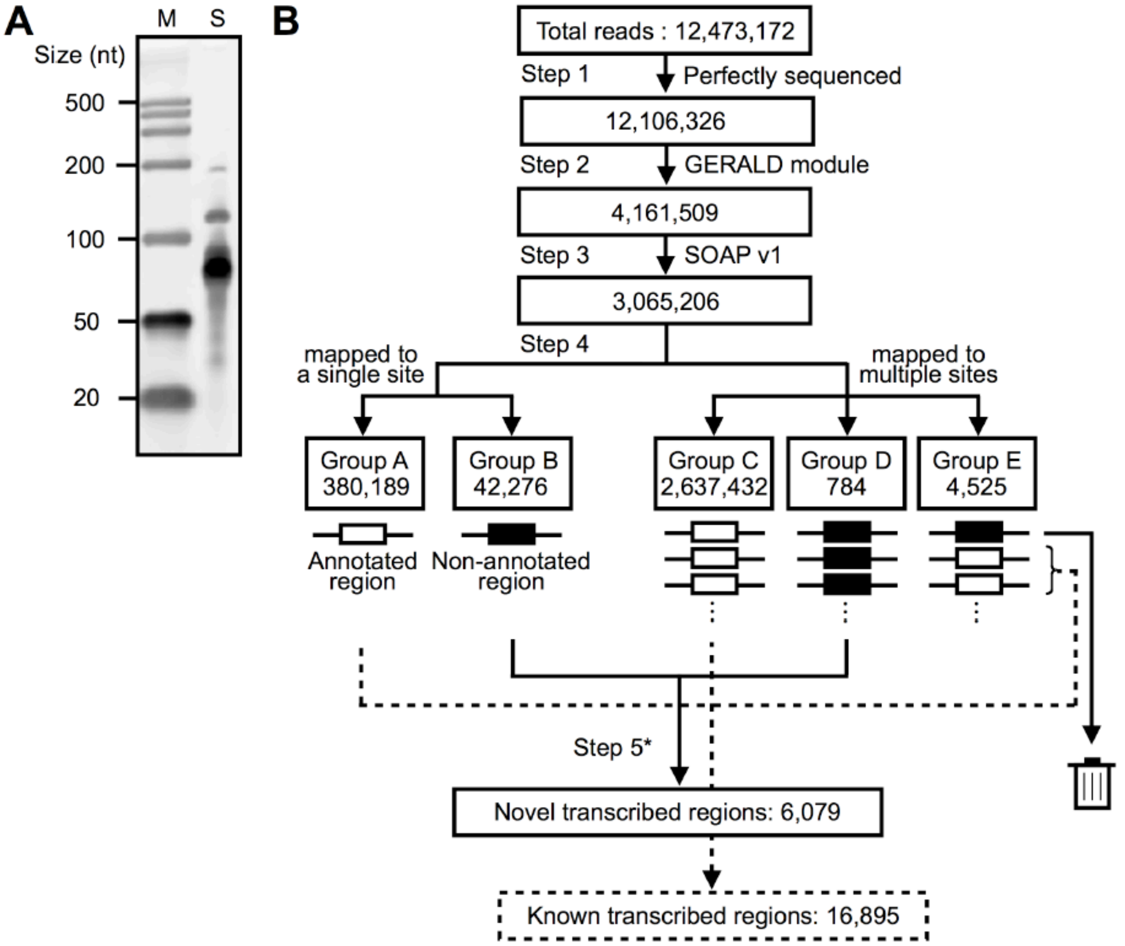

Additional File 1. Basic flow chart for the deep sequencing analysis of low-molecular-weight RNAs in *E. coli*. (A) Electrophoresis on a denaturing 6% polyacrylamide gel containing 8 M urea of the low-molecular-weight RNA fractions that were used for the deep sequencing analysis (lane S). The low-molecular-weight RNA was extracted from *E. coli* cells grown to late exponential phase. Lane M, RNA size marker (nt). (B) Prediction procedure for novel transcribed regions based on the deep sequencing data. Total reads were obtained from the deep sequencing analysis of low-molecular-weight RNA extracted from *E. coli* cells grown to late exponential phase. First, reads containing the character 'N' (indeterminable A, T, G or C) were discarded from the total reads (step 1). Second, reads with low-quality base calls were discarded based on the GERALD module (step 2). Third, we collected the reads that could be mapped to the *E. coli* genome using SOAP v1 (step 3). Fourth, the resulting reads were classified into five groups according to the numbers (single or multiple) and types (annotated or non-annotated) of the mapped regions (step 4). We eliminated the non-annotated mapped regions in Group E. Finally, all overlapping mapped regions were assembled, thus providing the novel and known transcribed regions (step 5). *After assembling the mapped regions in step 5, some non-annotated mapped regions in Groups B and D evidently belonged within the known transcribed regions.
