# Supplementary material for: Deep sequencing reveals as-yet-undiscovered small RNAs in Escherichia coli
Source: BMC Genomics. 2011 Aug 24;12:428. doi: 10.1186/1471-2164-12-428 (PMC3175480; doi:10.1186/1471-2164-12-428)
Supplement: Additional File 3 — Observed-to-expected (O/E) ratios for the sigma 70 promoter and rho-independent terminator for the novel transcribed regions. (A) The O/E ratios for the predicted sigma 70 promoter were calculated for the novel transcribed regions and for known genes with annotated sigma 70 promoters obtained from RegulonDB version 6.3, as the positive control. (B) The O/E ratios for the predicted rho-independent terminator were calculated for the novel transcribed regions and known genes with annotated rho-independent terminators obtained from RegulonDB version 6.3, as the positive control. [file 1471-2164-12-428-S3.PDF]

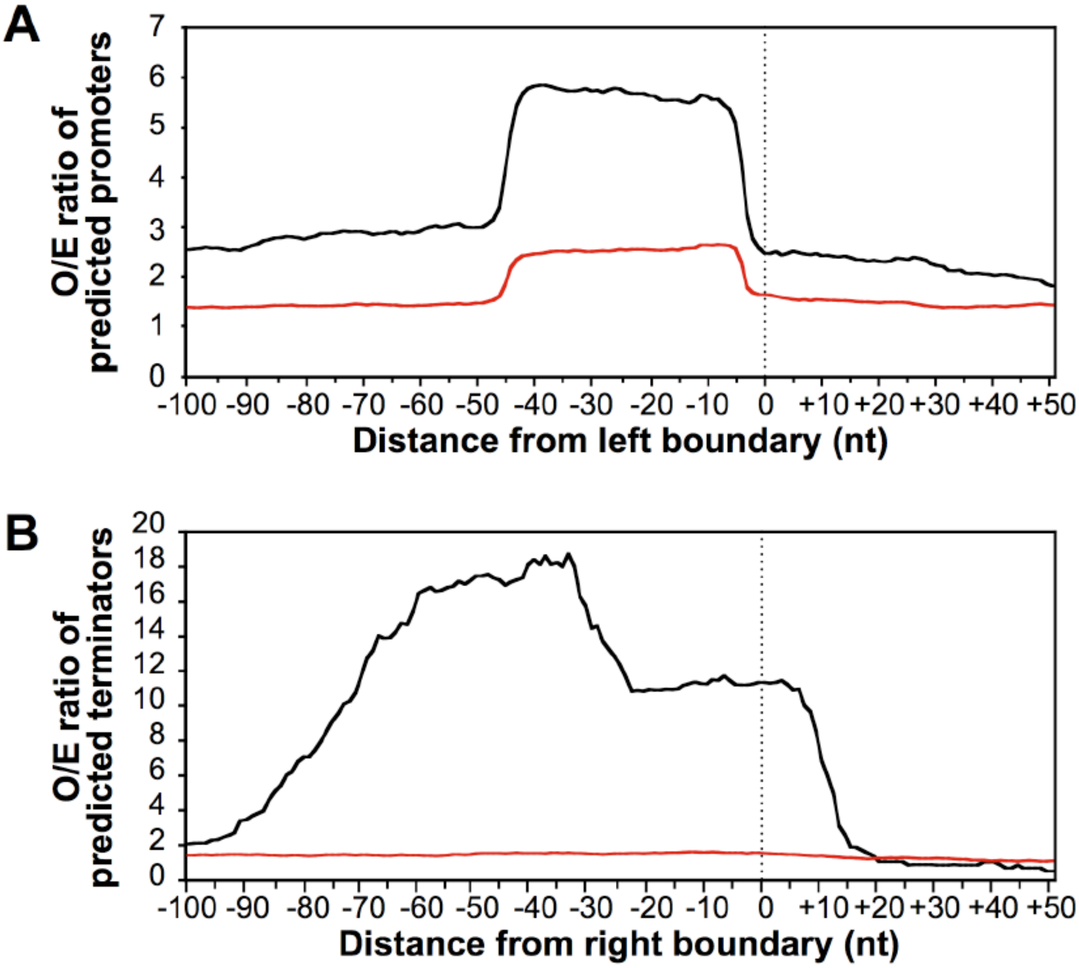

Additional File 3. Observed-to-expected (O/E) ratios for the sigma 70 promoter and rho-independent terminator for the novel transcribed regions. (A) O/E ratios for the predicted sigma 70 promoter were calculated (for details, see the Materials and Methods section) for the novel transcribed regions (red line, *n* = 6,079) and for known genes with annotated sigma 70 promoters obtained from RegulonDB version 6.3, as the positive control (black line, *n* = 741). The dotted line indicates the 5' end of each transcript. (B) The O/E ratios for the predicted rho-independent terminator were calculated (for details, see the Methods section) for the novel transcribed regions (red line, *n* = 6,079) and known genes with annotated rho-independent terminators obtained from RegulonDB version 6.3, as the positive control (black line, *n* = 187). The dotted line indicates the 3' end of each transcript.
