# Supplementary material for: Deep sequencing reveals as-yet-undiscovered small RNAs in Escherichia coli
Source: BMC Genomics. 2011 Aug 24;12:428. doi: 10.1186/1471-2164-12-428 (PMC3175480; doi:10.1186/1471-2164-12-428)
Supplement: Additional File 5 — Candidate sRNAs encoding putative small proteins. (A) Of the 229 candidate sRNAs, 159 were characterised by their small protein-encoding capacity. (B) Screen ECSBrowser shots for six sRNA regions encoding a putative small protein. [file 1471-2164-12-428-S5.PDF]

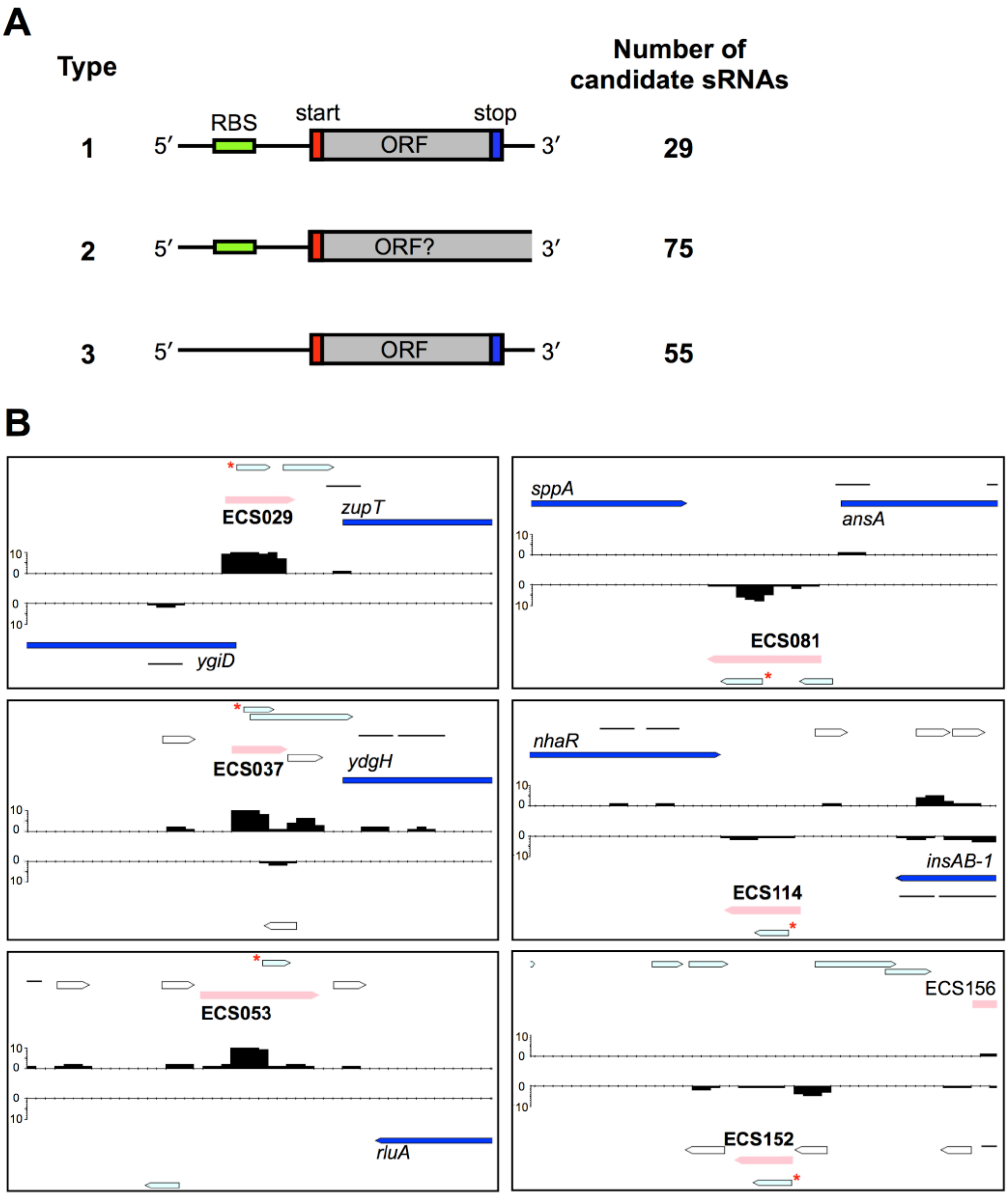

Additional File 5. Candidate sRNAs encoding putative small proteins. (A) Of the 229 candidate sRNAs (Figure 1B), 159 were characterised by their small protein-encoding capacity; They were classified into three types: those with a ribosomal binding site (RBS) and putative open reading frame (ORF) (type 1), those with an RBS and a start codon only (type 2); and those with an ORF without an RBS (type 3). (B) Screen ECSBrowser shots for six sRNA regions encoding a putative small protein. The pink boxed arrow indicates the position of the sRNA (this study). Note that these regions include possible peptide-encoding genes (light blue boxed arrows with asterisks) predicted by previous research (Hemm, M.R., Paul, B.J., Schneider, T.D., Storz, G. and Rudd, K.E. (2008) Small membrane proteins found by comparative genomics and ribosome binding site models. *Mol Microbiol*, **70**, 1487-1501).
