# Supplementary material for: Deep sequencing reveals as-yet-undiscovered small RNAs in Escherichia coli
Source: BMC Genomics. 2011 Aug 24;12:428. doi: 10.1186/1471-2164-12-428 (PMC3175480; doi:10.1186/1471-2164-12-428)
Supplement: Additional File 6 — Northern blot analysis confirming the growth-dependent expression of the ECS001, ECS005 and ECS007 sRNAs. Total RNA (20 μg per lane) was isolated from E. coli cells grown to an OD600 of 0.3, 0.6, 0.9 or 1.2 in M63 minimal medium. 5S rRNA expression is shown as the loading control. [file 1471-2164-12-428-S6.PDF]

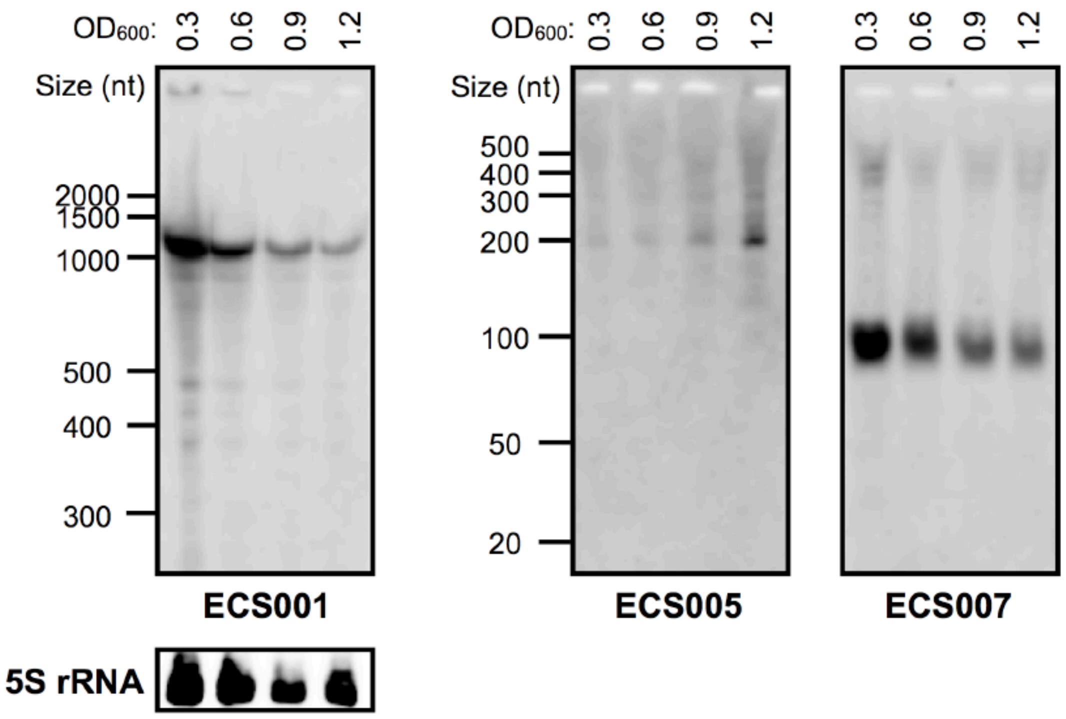

Additional File 6. Northern blot analysis confirmed the growth-dependent expression of the ECS001, ECS005 and ECS007 sRNAs. Total RNA (20 µg per lane) was isolated from *E. coli* cells grown to an OD_{600} of 0.3, 0.6, 0.9 or 1.2 in M63 minimal medium. 5S rRNA expression is shown as the loading control.
