# Supplementary material for: Deep sequencing reveals as-yet-undiscovered small RNAs in Escherichia coli
Source: BMC Genomics. 2011 Aug 24;12:428. doi: 10.1186/1471-2164-12-428 (PMC3175480; doi:10.1186/1471-2164-12-428)
Supplement: Additional File 7 — Growth of deletion mutants for six novel sRNAs. Wild type (WT, E. coli K12 strain BW25113) and single-deletion mutants corresponding to each intergenic sRNA region were grown overnight on either LB (rich medium) or M63 (glucose minimal medium) plates at 37, 42 or 20°C. These single-deletion mutants were systematically generated as described previously and the oligonucleotide primers used for the construction of these mutants are summarised in Additional File 8. [file 1471-2164-12-428-S7.PDF]

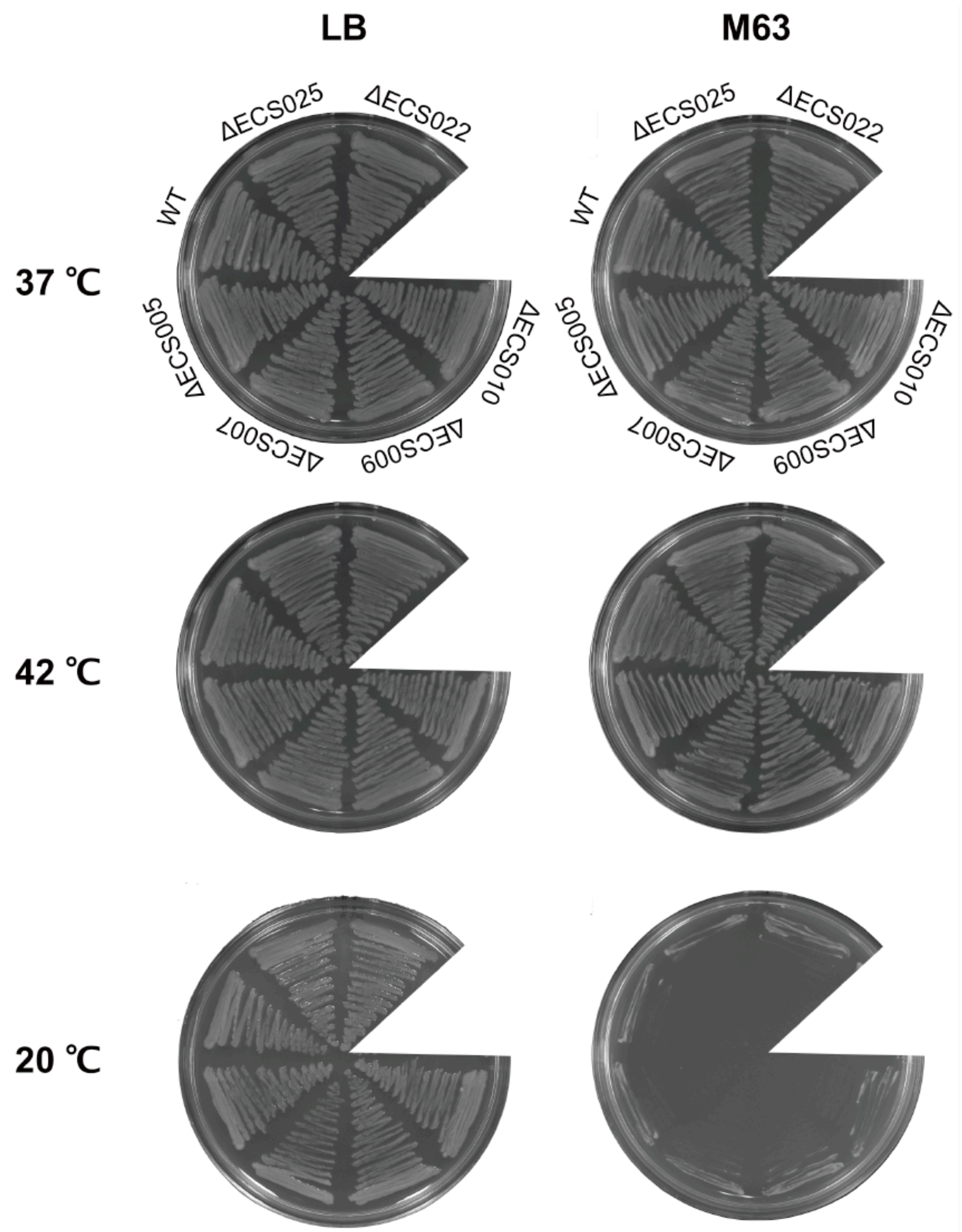

Additional File 7. Growth of deletion mutants for six novel sRNAs. Wild type (WT, *E. coli* K12 strain BW25113) and single-deletion mutants corresponding to each intergenic sRNA region were grown overnight on either LB (rich medium) or M63 (glucose minimal medium) plates at 37, 42 or 20 °C. These single-deletion mutants were systematically generated as described previously (Refs) and the oligonucleotide primers used for the construction of these mutants are summarised in Additional File 8.

References:

Baba, T., Ara, T., Hasegawa, M., Takai, Y., Okumura, Y., Baba, M., Datsenko, K.A., Tomita, M., Wanner, B.L. and Mori, H. (2006) Construction of *Escherichia coli* K-12 in-frame, single-gene knockout mutants: the Keio collection. *Mol Syst Biol*, **2**, 2006 0008.

Datsenko, K.A. and Wanner, B.L. (2000) One-step inactivation of chromosomal genes in *Escherichia coli* K-12 using PCR products. *Proc Natl Acad Sci U S A*, **97**, 6640-6645.
